# HEALTHCARE PROVIDERS’ EXPERIENCES IN CARING FOR OLDER ADULTS WITH MULTIPLE CHRONIC CONDITIONS

**DOI:** 10.1093/geroni/igz038.1160

**Published:** 2019-11-08

**Authors:** Jenny Ploeg, Marie-Lee Yous, Kimberly Fraser, Sinéad Dufour, Sharon Kaasalainen, Carrie McAiney, Maureen Markle-Reid, Lisa Garland Baird

**Affiliations:** 1 McMaster University, Hamilton, Ontario, Canada; 2 University of Alberta, Edmonton, Alberta, Canada; 3 University of Waterloo, Waterloo, Ontario, Canada; 4 University of Prince Edward Island, Charlottetown, Prince Edward Island, Canada

## Abstract

The management of multiple chronic conditions (MCC) in older adults living in the community is complex. Little is known about the experiences of interdisciplinary primary care and home providers who care for this vulnerable group. The aim of this study was to explore the experiences of healthcare providers in managing the care of community-living older adults with MCC and to highlight their recommendations for improving care delivery for this group. A qualitative interpretive description design was used. A total of 42 healthcare providers from two provinces in Canada participated in semi-structured interviews. Participants represented diverse disciplines (e.g., physicians, nurses, social workers, personal support workers) and settings (e.g., primary care and home care). Thematic analysis was used to analyze interview data. The experiences of healthcare providers managing care for older adults with MCC were organized into six major themes: (1) managing complexity associated with MCC, (2) implementing person-centred care, (3), involving and supporting family caregivers, (4) using a team approach for holistic care delivery, (5) encountering rewards and challenges in caring for older adults with MCC, and (6) recommending ways to address the challenges of the healthcare system. Healthcare providers highlighted the need for a more comprehensive integrated system of care to improve care management for older adults with MCC and their family caregivers. Specifically, they suggested increased care coordination, more comprehensive primary care visits with an interprofessional team, and increased home care support.

